# The endocycle regulators SIM and CCS52A1 control Arabidopsis root hair cell ploidy and expansion

**DOI:** 10.1093/plphys/kiaf352

**Published:** 2025-08-19

**Authors:** Lei Li (李磊), Klaus Herburger, Ilse Vercauteren, Duncan Coleman, Qian Wang, Steven Maere, Jefri Heyman, Lieven De Veylder

**Affiliations:** Department of Plant Biotechnology and Bioinformatics, Ghent University, Gent 9052, Belgium; Center for Plant Systems Biology, VIB, Gent 9052, Belgium; Institute of Biological Sciences, University of Rostock, 18051 Rostock, Germany; Department of Plant Biotechnology and Bioinformatics, Ghent University, Gent 9052, Belgium; Center for Plant Systems Biology, VIB, Gent 9052, Belgium; Department of Plant Biotechnology and Bioinformatics, Ghent University, Gent 9052, Belgium; Center for Plant Systems Biology, VIB, Gent 9052, Belgium; Institute of Biological Sciences, University of Rostock, 18051 Rostock, Germany; Department of Plant Biotechnology and Bioinformatics, Ghent University, Gent 9052, Belgium; Center for Plant Systems Biology, VIB, Gent 9052, Belgium; Department of Plant Biotechnology and Bioinformatics, Ghent University, Gent 9052, Belgium; Center for Plant Systems Biology, VIB, Gent 9052, Belgium; Department of Plant Biotechnology and Bioinformatics, Ghent University, Gent 9052, Belgium; Center for Plant Systems Biology, VIB, Gent 9052, Belgium

## Abstract

SIAMESE (SIM) and CELL CYCLE SWITCH 52 A1 (CCS52A1) control Arabidopsis root hair cell volume by controlling endoreplication.

Dear Editor,

Root hair (H) cells are specialized cells that enlarge the root–soil interface through tip growth, enhancing the uptake of water and nutrients (Grierson et al. 2014; Bienert et al. 2021). Interestingly, *Arabidopsis thaliana* (Arabidopsis) H cell development coincides with gain of polyploidy through endoreplication, being a specialized cell cycle variant in which the nuclear genome replicates without mitosis, resulting in a doubling of the DNA content with each cycle (Edgar et al. 2014). However, the relationship between endoreplication and H cell development is still unclear. DNA topoisomerase VI complex mutants, which fail to progress beyond the 8C ploidy level, have been found to display a disturbed root hair tip growth (Sugimoto-Shirasu et al. 2005; Kirik et al. 2007). Conversely, a diverse set of H cell developmental mutants display no clear correlation between cell length and endoreplication level (Sliwinska et al. 2015). This discrepancy may potentially be due to other processes than the endocycle being the primary cause of the root hair phenotype. Therefore, studying mutants in genes specifically affecting endoreplication may give a more conclusive understanding of the relationship between H cell growth and ploidy. Two endocycle regulators are SIAMESE (SIM) and CELL CYCLE SWITCH 52 A1 (CCS52A1), with the former being a cyclin-dependent kinase inhibitor and the latter an activating subunit of the anaphase-promoting complex/cyclosome resulting in cyclin degradation (De Veylder et al. 2011). Mutants of *SIM* and *CCS52A1* display a ploidy reduction in diverse tissues, including leaves and trichomes (Churchman et al. 2006; Vanstraelen et al. 2009; Breuer et al. 2012; Liu et al. 2012; Kumar et al. 2015; Schwarz and Roeder 2016). Here, we demonstrate that both genes control the H cell endocycle and we use the corresponding mutants to elucidate the function of endoreplication in root hair cell expansion.

First, we delineated the expression patterns of *SIM* and *CCS52A1* using cross-sections from the *SIM:GUS* and *CCS52A1:CCS52A1-GUS* reporter lines (see Supplementary Materials and Methods). Confirming previous findings (Takahashi et al. 2013; Bhosale et al. 2018), initial expression of both genes was observed in the basal meristem (BM) (Fig. 1, A and B). *SIM* first accumulated in H cells, but in the transition zone (TZ), its expression extended to the neighboring cells. *CCS52A1* was observed in all epidermal cells in the BM and spread into cortex cells in the TZ. For both genes, expression decreased in the elongation zone (EZ). The observed expression patterns of *SIM* and *CCS52A1* in the H cells suggested a potential involvement of both genes in the endoreplication process of those cells.

**Figure 1.**
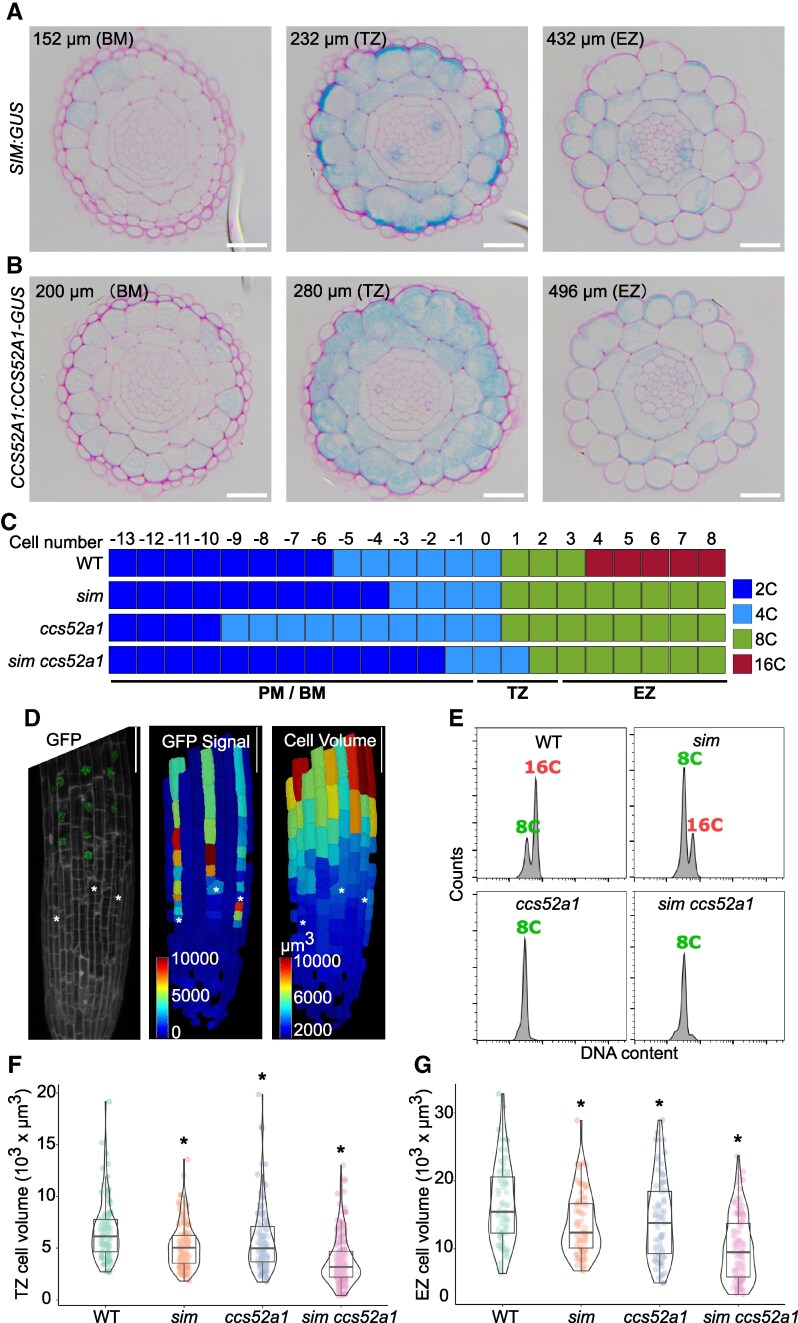
SIM and CCS52A1 determine the endoreplication level of H cells in the Arabidopsis root. **A** and **B)** Cross-sections following β-glucuronidase staining at the indicated distance of the root tip of 5-d-old roots of *SIM:GUS*  **A)** and *CCS52A1:CCS52A1-GUS*  **B)** seedlings, corresponding to the basal meristem (BM), transition zone (TZ), and elongation zone (EZ). Cells are outlined through ruthenium red staining. Scale bars, 20 *μ*m. **C)** Mapping of H cell DNA ploidy levels along the BM, TZ, and EZ of WT (Col-0) and *sim*, *ccs52a1*, and *sim ccs52a1* roots based on DAPI staining. The first elongated cell is set as the “0” point, marking the start of the TZ. Data were obtained from 4 to 5 independent roots. **D)** Representative confocal image of an *AT2G34910:H2A-GFP* root counterstained with propidium iodide (left panel), GFP expression heat map (middle panel), and cell volume heat map (right panel). MorphoGraphX was used to digitalize the images. The white asterisks indicate the H cells showing the first GFP signal. The colored bars indicate the value of GFP fluorescence (middle panel) and cell volume (right panel). Scale bars, 50 *μ*m. **E)** Ploidy distribution of *AT2G34910:H2A-GFP* positive cells in the 7-d-old roots of WT (Col-0), *sim*, *ccs52a1,* and *sim ccs52a1* roots based on dual-color flow cytometry. **F** and **G)** H cell volume of WT (Col-0) and mutants in the TZ **F)** and EZ **G)**. The center lines in the boxplots show the median, with the box limits representing the upper and lower quartiles and the whiskers representing the maximum and minimum values. Dots drawn outside the whiskers mark outliers outside 1.5 × the interquartile range above or below the box. The asterisks indicate a significant difference (adjusted *P* < 0.05) determined by nonparametric 1-way ANOVA followed by Dunnett's test post hoc test, compared to the WT (*n* ≥ 60 cells per genotype).

To analyze the effects of SIM and CCS52A1 on H cell endoreplication, we mapped the DNA ploidy levels by means of DAPI staining in *sim* and *ccs52a1* single mutants and the *sim ccs52a1* double mutant. We quantified the DNA ploidy level from the 13 last H cells within the meristem up to the 8 earliest cells leaving the meristematic zone, covering the complete TZ (Fig. 1C; Supplementary Fig. S1). In wild-type (WT) plants, H cells obtained a 4C DNA content before entering the TZ, suggesting that cells enter the endocycle before rapid cell elongation (Fig. 1C). Within the TZ, cells underwent an extra endocycle, reaching 8C. A third endocycle was observed within the EZ, resulting in a 16C ploidy content, confirming previous mathematical modeling data (Bhosale et al. 2018). Differently from WT, both *sim* and *ccs52a1* single mutants did not show the presence of 16C cells in the EZ (Fig. 1C), and thus, H cells remained at the 8C stage. To validate this, we constructed a marker line with an H cell-specific promoter driving a histone *H2A-GFP* reporter gene (*AT2G34910:H2A-GFP*; Supplementary Fig. S2). H2A-GFP fluorescence was observed in the BM, preceding the strong increase in cell volume (Fig. 1D), but predominantly marked cells of the TZ and EZ (Supplementary Fig. S2). Accordingly, dual-color flow cytometry confirmed the DNA ploidy levels of the GFP-positive cells within the elongating WT H cells to be 8C or 16C (Fig. 1E). Differently, *sim* H cells showed a strong decrease in 16C cells, while *ccs52a1* and *sim ccs52a1* H cells remained at the 8C stage (Fig. 1E). MorphoGraphX was used to investigate the effects on epidermal cell growth, revealing a reduction in cell volume for all mutants in both the TZ and EZ, being most outspoken in the double mutant (Fig. 1, F and G).

Following entry in the EZ, H cell tip growth is initiated. To investigate the function of SIM and CCS52A1 in this process, we first examined the endoreplication levels of the WT and the different mutants using the *AT1G27740:H2A-GFP* reporter that is specifically expressed in the tip growth-initiating cells (Supplementary Fig. S2). Dual-color flow cytometry showed that the H cells from 7-d-old WT roots accumulated a ploidy level of predominantly 16C, whereas the *sim* and *ccs52a1* mutants displayed reduced ploidy levels in H cells, indicated by the increase of the 8C-containing cell population (Fig. 2A). The *sim ccs52a1* double mutant exhibited an enhanced endoreplication defect compared to the single mutants, with the 16C population being barely detectable (Fig. 2A). These findings demonstrate that SIM and CCS52A1 redundantly control the 8C-to-16C transition of maturing H cells. To assess the effects of SIM and CCS52A1 on root hair tip growth, root hair parameters were measured. Within the *ccs52a1* mutant, the first visual tip-grown cell was found more distantly from the root tip compared to the WT (Fig. 2, B and C), indicative for a delayed hair tip cell differentiation. In all mutants, the final root hair length was significantly reduced compared to the WT (Fig. 2D). In addition, time-lapse imaging showed that the root hair growth rate was significantly decreased in the *ccs52a1* and *sim ccs52a1* mutants compared to both WT and *sim* (Fig. 2E), indicating a positive effect of CCS52A1 on the root hair growth rate.

**Figure 2.**
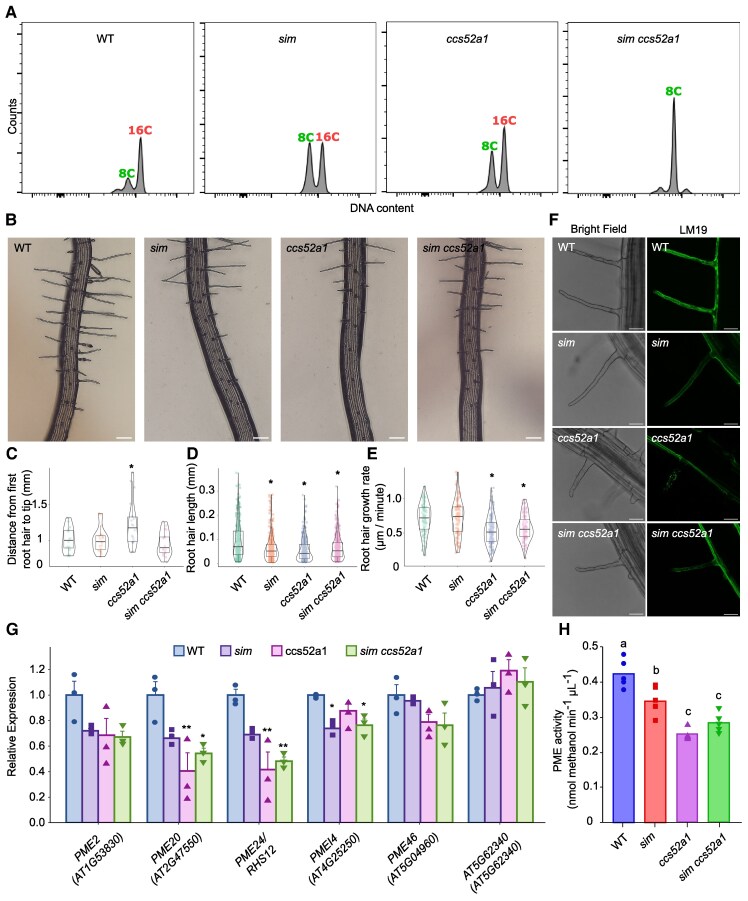
Impact of *sim* and *ccs52A1* mutations on the ploidy level and volume of tip-growing H cells. **A)** Ploidy distribution of *AT1G27740:H2A-GFP* positive cells in 7-d-old roots of WT (Col-0), *sim*, *ccs52a1,* and *sim ccs52a1* mutants based on dual-color flow cytometry. **B)** Representative root images used for quantitative measurements in **C)** to **E)** of WT (Col-0), *sim*, *ccs52a1,* and *sim ccs52a1*. Scale bars, 0.1 mm. **C** to **E)** Quantitative measurements of the distance from the root tip to the first visible root hair **C)**, root hair length **D)**, and root hair growth rate **E)**. Data were obtained from over 15 independent roots per genotype (*n* ≥ 80 cells per genotype). The center lines in the boxplots show the median, with the box limits representing the upper and lower quartiles and the whiskers representing the maximum and minimum values. Dots drawn outside the whiskers mark outliers outside 1.5 × the interquartile range above or below the box. Asterisks indicate significant differences (adjusted *P* < 0.05) between WT and mutants. For root hair length and growth rate, the statistical analysis was done by nonparametric 1-way ANOVA, followed by Dunnett's post hoc test; for distance from the first root hair to tip, the statistical analysis was done by parametric 1-way ANOVA, followed by Dunnett's post hoc test. **F)** Bright-field and fluorescence images of root hairs of WT (Col-0) and *sim*, *ccs52a1*, and *sim ccs52a1* mutants stained with the monoclonal antibody LM19 (green fluorescence). Scale bars, 50 *µ*m. **G)** Relative expression levels of *PME/PMEI* genes in 7-d-old root tips of WT (Col-0), *sim*, *ccs52a1*, and *sim ccs52a1*. Expression of the WT was arbitrarily set to 1. Bars represent average ± standard error. Asterisks indicate significant differences (*P < 0.05) compared to the WT, determined by parametric 1-way ANOVA and Tukey post hoc test (*n* = 3 biological repeats per genotype). **H)** PME activity in crude root tip extracts. Small letters indicate statistical differences (*P* < 0.05) among groups, determined by parametric 1-way ANOVA followed by Tukey's post hoc test (*n* = 5 biological repeats per genotype).

During cell expansion, the plant cell wall is subjected to modifications. In light of the observed decrease in the volume of H cells in the primary roots of the *sim* and *ccs52a1* mutants, we investigated potential modifications in the primary root cell wall by employing the comprehensive microarray polymer profiling method (Moller et al. 2012; Rydahl et al. 2018). Statistical analysis showed in the *sim*, *ccs52a1*, and *sim ccs52a1* mutants a significant reduction of (1→5)-α-L-arabinan (LM6), extensins (JIM12, JIM20, and LM3), and homogalacturonan (HG) with a low degree of methylesterification (DE) (recognized by probes LM18 and LM19), compared to the WT (Supplementary Fig. S3 and Table S1). The latter was confirmed in root hairs by immunolocalization staining using the LM19 antibody (Fig. 2F), suggesting that SIM- and CCS52A1-mediated endoreplication may affect the demethylesterification pattern of HG. The HG demethylesterification pattern is determined by the interplay between *pectin methyl esterase* (*PME*) and *PME inhibitor* (*PMEI*) genes. To test whether the observed reduction of HG demethylesterification within the H cells may originate from an altered expression of *PME/PMEI* genes, we quantified the transcript level of a selection of *PME/PMEI* genes showing a high transcript abundance in WT H cells. Among the genes tested, a significant reduction in *PME20*, *PME24/RHS12*, and *PMEI4* transcript levels was observed within the different mutant roots, compared to the WT (Fig. 2G). The decreased *PME* transcript levels were corroborated by biochemical data, illustrating a significant decrease of PME activity by approximately 18% to 40% in *sim*, *ccs52a1*, and *sim ccs52a1* mutants, when compared to that of the WT (Fig. 2H).

In conclusion, we found a close relationship between cell volume and ploidy level for Arabidopsis root H cells, with H cells gaining a 4C and 16C ploidy before the observed rapid cell volume increase in the EZ and the initiation of root hair tip growth, respectively. Moreover, both *sim* and *ccs52a1* mutants displayed a decreased TZ and EZ cell volume, which was enhanced in the double mutant. These data strongly support a contribution of endoreplication to cell growth. The mechanism by which SIM and CCS52A1 regulate H cell expansion during endoreplication remains unclear, but the observed effects on PMEs combined with other reports (Bhosale et al. 2019; Ma et al. 2022; Goldy et al. 2023) suggest that endoploidy changes relate to cell wall modifications that may influence final cell size. The mechanistic framework behind this ploidy change-induced cell wall modification remains to be revealed.

## Supplementary Material

kiaf352_Supplementary_Data

## Data Availability

The data underlying this article are available in the article and in its online supplementary material.

## Supplementary data

The following materials are available in the online version of this article.

**Supplementary Figure S1.** Mapping of ploidy borders in H cell files.

**Supplementary Figure S2.** Expression patterns of *AT2G34910:H2A-GFP* and *AT1G27740:H2A-GFP* root hair cell marker lines.

**Supplementary Figure S3.** Impact of *sim* and *ccs52A1* mutations on primary root cell wall modifications.

**Supplementary Table S1.** Carbohydrate microarray on different extractions of WT and mutant roots.

**Supplementary Table S2.** Arabidopsis lines used in this study.

**Supplementary Table S3.** Sequences of primers used for generating root hair-specific marker lines.

**Supplementary Table S4.** Sequences of primers used for RT-qPCR analysis.

## Dive Curated Terms

The following phenotypic, genotypic, and functional terms are of significance to the work described in this paper:

SIM Gramene: AT5G04470
SIM Araport: AT5G04470

DAPI CHEBI: CHEBI:51231

root AmiGo: PO:0009005

CCS52A1 Gramene: At4g22910
CCS52A1 Araport: At4g22910

AT2G34910:H2A-GFP Gramene: AT2G34910
AT2G34910:H2A-GFP Araport: AT2G34910
