# Supplementary material for: The endocycle regulators SIM and CCS52A1 control Arabidopsis root hair cell ploidy and expansion
Source: Plant Physiol. 2025 Aug 19;198(4):kiaf352. doi: 10.1093/plphys/kiaf352 (PMC12362249; doi:10.1093/plphys/kiaf352)
Supplement: kiaf352_Supplementary_Data [file kiaf352_supplementary_data.pdf]

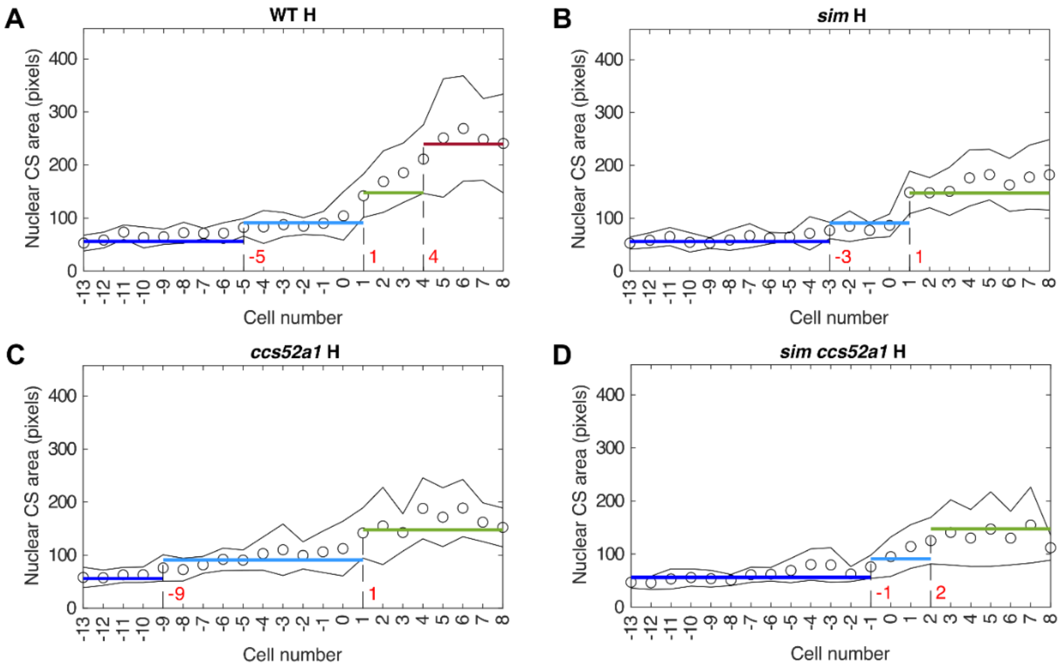

Supplementary Figure S1. Mapping of ploidy borders in H cell files.

Panels show the optimal ploidy profile fit, across 500 optimization runs, of a piecewise linear model (see Methods) to cross-sectional nuclear area data of wild type (WT, A), *sim* (B), *ccs52a1* (C), and *sim ccs52a1* (D) H cell files. Open circles indicate the average nuclear cross-sectional area in pixels across all cells of a given rank, and black lines are located one standard deviation above and below the average. The fitted average nuclear cross-sectional areas for 2C, 4C, 8C and 16C cells are indicated with dark blue, light blue, green and red line pieces, respectively. Cell numbers where the fitted ploidy profile shifts are indicated in red font.

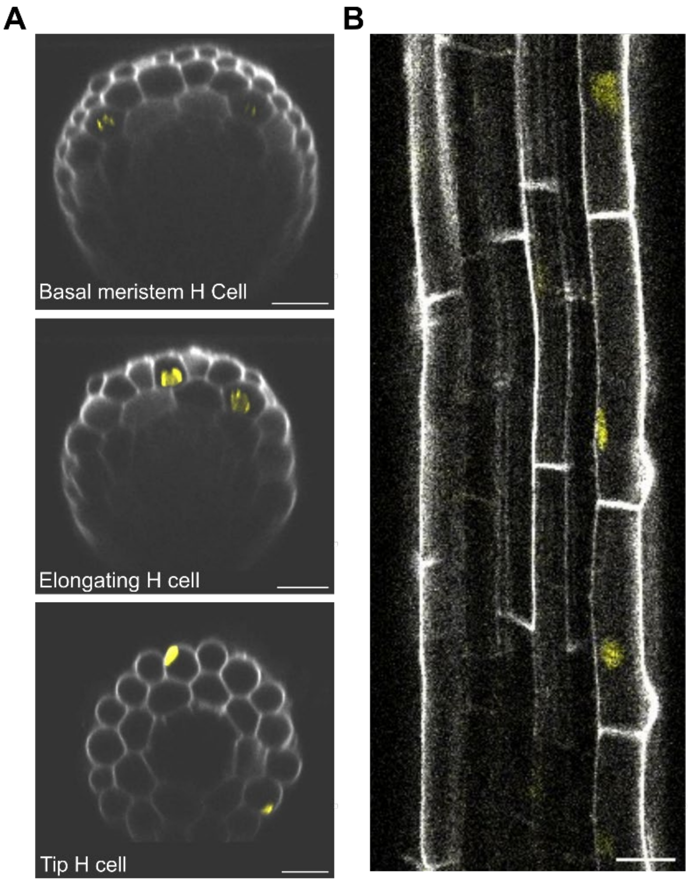

Supplementary Figure S2. Expression patterns of *AT2G34910:H2A-GFP* and *At1g27740:H2A-GFP* root hair cell marker lines. A, *AT2G34910:H2A-GFP* confocal microscope root cross images through meristematic (top), elongating (middle), and tip (bottom) H cells. **B,** *At1g27740:H2A-GFP* reporter confocal microscope longitudinal image of a tip growing cell. Cell walls were visualized by propidium iodide staining (white). Scale bars, 20 µm.

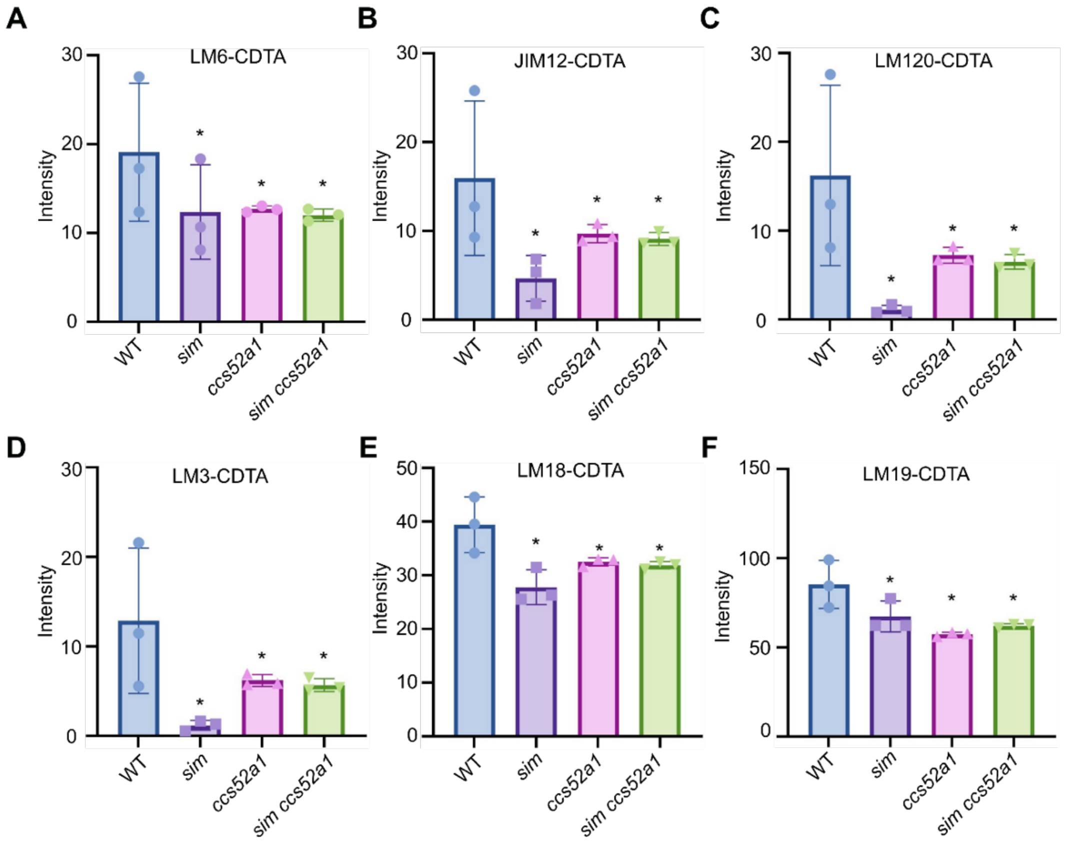

Supplementary Figure S3. Impact of *sim* and *ccs52A1* mutations on primary root cell wall modifications. A-F, Quantification of the intensity of the LM6 (A), JIM12 (B), LM120 (C), LM3 (D), LM18 (E) and LM19 (F) signal in cell wall extracts of 7-day-old roots of WT (Col-0), *sim*, *ccs52a1* and *sim ccs52a1*. Asterisks denote significant differences compared to the control ($p < 0.05$) determined by two-way ANOVA followed by Dunnett's post-hoc test (n = 6 pools of 2 biological repeats per genotype).

Supplementary Table S1. Carbohydrate microarray on different extractions of WT and mutant roots.

Pectin and hemicellulose were extracted using 1,2-cyclohexylenedinitrilotetraacetic acid (CDTA) and NaOH, respectively. The spot value represents the signal of intensity of each immune antibody, with the higher intensity of green coloration indicating higher values. All spot intensity values are normalized to the highest fluorescence value. n = 6 pools of 2 biological repeats. DE, degree of methylesterification. Statistically significant differences between groups are indicated by small letters (CDTA extracts) or capital letters (NaOH extracts); they were determined with a nonparametric one-way ANOVA followed by Tukey's postdoc test.

			CDTA				NaOH			
**Category**	**Probe name**	**Epitope**	**Col-0**	***sim***	***ccs52a1***	***sim ccs52a1***	**Col-0**	***sim***	***ccs52a1***	***sim ccs52a1***
**Pectins**	**2F4**	Ca^{2+} cross linked homogalacturonan	18^{a}	16^{a}	17^{a}	17^{a}	0^{A}	0^{A}	0^{A}	0^{A}
	**JIM5**	Homogalacturonan with a low DE	16^{a}	11^{a}	12^{a}	12^{a}	0^{A}	0^{A}	0^{A}	0^{A}
	**JIM7**	Homogalacturonan with a high DE	5^{a}	2^{a}	4^{a}	3^{a}	0^{A}	0^{A}	0^{A}	0^{A}
	**LM18**	Methylesterified homogalacturonan (low DE)	39^{a}	28^{bc}	33^{ab}	32^{ab}	20^{A}	13^{A}	16^{A}	15^{A}
	**LM19**	Methylesterified homogalacturonan (low DE)	85^{a}	67^{ab}	57^{bc}	62^{bc}	30^{A}	29^{A}	26^{A}	26^{A}
	**LM20**	Methylesterified homogalacturonan (high DE)	2^{a}	2^{a}	4^{a}	3^{a}	2^{A}	3^{A}	3^{A}	4^{A}
	**INRA-RU2**	Backbone of rhamnogalacturonan I (4 units)	21^{a}	30^{a}	26^{a}	30^{a}	5^{A}	14^{A}	11^{A}	10^{A}
	**INRA-RU1**	Backbone of rhamnogalacturonan I (12 units)	13^{a}	20^{a}	19^{a}	18^{a}	0^{A}	5^{B}	3^{B}	3^{B}
	**CCRC-M1**	RG I (specificity lower than RU2/1)	16^{a}	24^{b}	22^{b}	11^{a}	28^{A}	31^{A}	31^{A}	29^{A}
	**CCRC-M13**	RG I (specificity lower than RU2/1)	0^{a}	0^{a}	0^{a}	0^{a}	0^{A}	0^{A}	0^{A}	0^{A}
	**LM5**	(1→4)-β-D-galactan	7^{a}	5^{a}	4^{a}	4^{a}	3^{A}	2^{A}	3^{A}	2^{A}
	**LM6**	(1→5)-α-L-arabinan	19^{a}	12^{b}	13^{b}	12^{b}	3^{A}	4^{A}	4^{A}	3^{A}
	**LM13**	Linearized (1→5)-α-L-arabinan	0^{a}	0^{a}	0^{a}	0^{a}	0^{A}	0^{A}	0^{A}	0^{A}
	**LM16**	(1→5)-α-L-arabinan, RG backbone	4^{a}	0^{a}	2^{a}	2^{a}	2^{A}	1^{A}	1^{A}	1^{A}
	**LM8**	Various types of xylogalacturonans	0^{a}	0^{a}	0^{a}	0^{a}	0^{A}	0^{A}	0^{A}	0^{A}
**Mannans**	**BS-400-4**	(1→4)-β-D-mannan and galacto-(1→4)-β-D-mannan	0^{a}	0^{a}	0^{a}	0^{a}	1^{A}	2^{A}	1^{A}	1^{A}

			CDTA				NaOH			
**Category**	**Probe name**	**Epitope**	**Col-0**	***sim***	***ccs52a1***	***sim ccs52a1***	**Col-0**	***sim***	***ccs52a1***	***sim ccs52a1***
	**LM21**	(1→4)-β-D-mannan/galactomannan/glucomannan	0^{a}	0^{a}	0^{a}	0^{a}	14^{A}	16^{AB}	22^{B}	22^{B}
	**LM22**	(1→4)-β-D-mannan/glucomannan	0^{a}	0^{a}	0^{a}	0^{a}	0^{A}	0^{A}	0^{A}	0^{A}
**Xyloglucans**	**CCRC-M58**	Xyloglucan (from tamarind)	0^{a}	0^{a}	0^{a}	0^{a}	35^{A}	42^{A}	40^{A}	41^{A}
	**LM15**	Xyloglucan (XXXG motif)	1^{a}	7^{a}	5^{a}	6^{a}	48^{A}	63^{B}	61^{B}	59^{B}
	**LM24**	Galactosyl residues of xyloglucan	0^{a}	0^{a}	0^{a}	0^{a}	0^{A}	1^{A}	0^{A}	0^{A}
	**LM25**	Xyloglucan (XXXG, XXLG, XLLG motif; also, GGGGGG)	3^{a}	6^{a}	7^{a}	8^{a}	51^{A}	59^{A}	62^{A}	63^{A}
**Xylans**	**LM10**	(1→4)-β-D-xylan (low-substituted)	1^{a}	0^{a}	0^{a}	0^{a}	14^{A}	13^{A}	14^{A}	13^{A}
	**LM11**	(1→4)-β-D-xylan (low-substituted)/arabinoxylan	1^{a}	0^{a}	1^{a}	1^{a}	22^{A}	17^{A}	17^{A}	17^{A}
	**LM23**	(1→4)-β-D-xylan/xylogalacturonan	1^{a}	0^{a}	0^{a}	0^{a}	2^{A}	0^{A}	1^{A}	1^{A}
	**LM28**	Glucuronoxylan	5^{a}	2^{a}	3^{a}	3^{a}	43^{A}	42^{A}	43^{A}	42^{A}
	**CCRC-M39**	4-O-methylglucuronoxylan	0^{a}	0^{a}	0^{a}	0^{a}	41^{A}	44^{A}	44^{A}	43^{A}
**β-glucans**	**BS-400-2**	(1→3)-β-D-glucan	1^{a}	0^{a}	0^{a}	0^{a}	4^{A}	11^{B}	12^{B}	11^{B}
	**BS-400-3**	(1→3)(1→4)-β-D-glucan	0^{a}	0^{a}	0^{a}	0^{a}	3^{A}	0^{A}	1^{A}	1^{A}
**Arabino-galactan-proteins (AGPs)**	**JIM8**	AGP (Gal-rich)	0^{a}	0^{a}	0^{a}	0^{a}	0^{A}	0^{A}	0^{A}	0^{A}
	**JIM13**	AGP (β-GlcA-(1 → 3)-α-GalA-(1 → 2)-α-Rha)	9^{a}	2^{a}	5^{a}	5^{a}	14^{A}	5^{B}	9^{AB}	9^{AB}
	**LM2**	AGP (1 → 6)-β-Gal with terminal β-GlcA)	1^{a}	0^{a}	1^{a}	1^{a}	15^{A}	1^{B}	7^{AB}	7^{AB}
	**LM14**	AGP, enriched Ara, Gal and aldouronic acid	8^{a}	1^{a}	4^{a}	4^{a}	25^{A}	11^{B}	16^{B}	15^{B}
	**MAC207**	AGP (β-GlcA-(1 → 3)-α-GalA-(1 → 2)-α-Rha)	4^{a}	1^{a}	2^{a}	2^{a}	14^{A}	4^{B}	8^{AB}	8^{AB}
**Extensins**	**LM1**	Extensin (hydroxyproline-rich motif THRGP)	1^{a}	0^{a}	1^{a}	1^{a}	0^{A}	0^{A}	0^{A}	0^{A}
	**LM3**	Extensin	13^{a}	1^{b}	6^{ab}	6^{ab}	5^{A}	1^{A}	3^{A}	2^{A}
	**JIM11**	Extensin	14^{a}	1^{b}	6^{ab}	6^{ab}	14^{A}	3^{B}	8^{AB}	8^{AB}
	**JIM12**	Extensin	16^{a}	5^{a}	10^{a}	9^{a}	9^{A}	2^{B}	5^{AB}	5^{AB}
	**JIM19**	Extensin	0^{a}	0^{a}	0^{a}	0^{a}	0^{A}	0^{A}	0^{A}	0^{A}
	**JIM20**	Extensin	16^{a}	1^{b}	7^{ab}	7^{ab}	21^{A}	5^{B}	12^{AB}	12^{B}
**Cellulose**	**CBM3a**	Crystalline cellulose	2^{a}	1^{a}	2^{a}	2^{a}	2^{A}	3^{A}	3^{A}	3^{A}
	**CBM2a**	Crystalline cellulose	1^{a}	1^{a}	1^{a}	1^{a}	42^{A}	42^{A}	43^{A}	44^{A}

Supplementary Table S2. Arabidopsis lines used in this study.

Line	Description
sim	Bhosale et al., 2018
ccs52a1-1	Salk_083656
sim ccs52a1	Crossed line
SIM:GUS	Bhosale et al., 2018
CCS52A1:CCS52A1-GUS	Willems et al., 2020
At2G34910:H2A-GFP	This study
At1g27740:H2A-GFP	This study

Supplementary Table S3. Sequences of primers used for generating root hair-specific marker lines.

Promoter	Length (bp)	Description	Sequence (5' to 3')
At2G34910	2148	Forward	**AGAAGTGAAGCTTGGTCTCAACCT**TAAGGTCAGGGCTGGAAACA
		Reverse	**AGGGCGAGAATTCGGTCTCATGTT**AACAAAGTCAGAAATCTTAA
At1G27740	1434	Forward	**AGAAGTGAAGCTTGGTCTCAACCT**ATACATGCATGAATAACGAA
		Reverse	**AGGGCGAGAATTCGGTCTCATGTT**TAACTTATTTGGATGAAGCT

The bold bases represent the overhang with module A-B entry vectors.

Supplementary Table S4. Sequences of primers used for RT-qPCR analysis.

Gene	Oligo Name	Sequence
At1g02780	EMB_F	CTCTCGTTCCAGAGCTCGCAAAA
	EMB_R	AAGAACACGCATCCTACGCATCC
At3g22110	PAC_F	TCTCTTTGCAGGATGGGACAAGC
	PAC_R	AGACTGAGCCGCCTGATTGTTTG
At5G62340	AT5G62340_F	GTTGGACCGACTGCTTGTTT
	AT5G62340_R	TCGACGCTATGACTCTCACC
At5G04960	PME46_F	GATCTTGCCTCGTCGTCCTA
	PME46_R	TCCTGGCCCGGAATTTAAGT
At4G25250	PMEI4 _F	TTTCCAAAGACAGCAGCCAC
	PMEI4 _R	ATTGGGTCGGACTTGATGGT
At3G10710	PME24_F	CGGTTCCTGAGCTAGTCGAT
	PME24_R	CTTCGCCACCACAATATCCG
At2G47550	PME20_F	ACTGTCGTTATCTAGGGCCG
	PME20_R	GGATCACCGTCACAATGTCG
At1G53830	PME2_F	ACCAATGCGACATGTTTGCT
	PME2_R	AGTGTCCAACGCAAAACTCC

Supplementary materials and methods

Plant material, growth conditions and chemical treatments

Arabidopsis thaliana lines (Supplemental Table S2) used are in the Columbia-0 (Col-0) background. Sterilized seeds were plated on a sugar-free 1/2 Murashige and Skoog (MS) medium supplemented with 1 % plant agar and 0.5 g/L MES, adjusted to pH 5.8. The seeds were stratified in the dark at 4 °C for three days and then transferred to a growth room with a 16 h light/8 h dark cycle (70 µmol $m^{-2}s^{-1}$) at 22 °C.

The transgenic lines *At2g34910:H2A-GFP* and *At1g27740:H2A-GFP* expressing the H2A-GFP fusion protein in the H files were generated using GreenGate system, a modular plasmid construction method based on Golden Gate cloning, as described by Decaestecker et al. (2019). Five entry modules [pGG A-promoter-B (containing a tissue-specific promoter), pGG B-linker-C (encoding a linker), pGG C-H2A-D (encoding histone H2A without a stop codon), pGG D-GFP-E (encoding the GFP fluorescence tag), and the pGG E-terminator-F (containing the G7 terminator) were assembled into a destination pFASTR-A-G in a single reaction using the restriction enzyme BsaI and T4 DNA ligase. The primer sequences used to amplify and clone the promoter regions into the pGG A-promoter-B module are provided in Supplemental Table S2. Imaging of the reporter lines was performed using an optical chamber using an

LSM 900 confocal laser-scanning microscope (Zeiss) equipped with a ×20 air objective lens (NA 0.8). Cell outlines were obtained by staining with 2μg/mL propidium iodide (PI) for 5 min, then washed with water. PI was excited at 535 nm, with emission detected at 617 nm. For eGFP, excitation occurred at 488 nm, with emission collected using a 500–530 nm bandpass filter.

3D analysis by MorphoGraphX

High-resolution images were acquired using an LSM 710 confocal laser-scanning microscope (Zeiss) equipped with a ×40 water immersion objective lens (NA 0.8). Cell outlines were obtained by staining with Calcofluor white using the ClearSee protocol (Kurihara et al., 2015). The acquired confocal images were converted to TIFF format using ImageJ software for subsequent 3D segmentation with MorphoGraphX (version 2.0) (Montenegro-Johnson et al., 2015; Wolny et al., 2020). To enhance image quality, the images were smoothed using a Gaussian blur (x=0.6, y=0.6, z=0.3) and then segmented with ITK=1000. Manual corrections were performed to refine segmentation mistakes. The 3D cellular mesh was created with a cube size of 1.0. Geometrical parameters were analyzed using the "heatmap" function. For position analysis of the roots of the WT and the *sim*, *ccs52a1*, and *sim ccs52a1* mutants, the "3DCellAtlas" function of MorphoGraphX (Strauss et al., 2022) was applied. This involved the creation of a 2.5D surface mesh. Mapping analysis was performed using the "ggstatsplot" R package.

Endoploidy mapping analysis

The nuclei from 7-day-old roots were stained with DAPI solution to quantify DNA content, and cell outlines were stained with SR2200 (Musielak et al., 2016; Tofanelli et al., 2019). Optical sections were acquired in Z stacks using an LSM710 confocal microscope (Zeiss). DAPI-stained nuclear cross-sectional area (in pixels) was used as a proxy for cell ploidy level. Nuclear cross-sectional areas were quantified from the central optical sections of targeted nuclei using ImageJ software. For analysis, nuclei were sampled from 8-10 H files across 4–5 independent seedlings per genotype. A piecewise linear model was fit to the nuclear cross-sectional area data across cell files and genotypes using Matlab R2024a. The last cell of all cell files was not modeled because these cells often exhibited unusually low nuclear cross-sectional area values, likely due to imaging bias. The first and second-last cells in the imaged cell range (cell ranks -13 and 8) have fewer data points for several genotype/cell type combinations due to missing data, leading to very low or zero standard deviations. In order not to overly restrict model optimization (see below), the standard deviation for a cell with <4 data points in a particular genotype/cell type combination was set to the standard deviation of the following cell (for the first cell) or the preceding cell (for the second-last cell). The 26 parameters in the model are the average nuclear cross-sectional area of 2C cells, a scaling factor that models how the average nuclear cross-sectional area scales with ploidy, and the average 2C-4C, 4C-8C and 8C-16C boundaries along the cell file and

genotype (WT, *sim*, *ccs52a1* and *sim ccs52a1*). All parameters were estimated from the data. The 'particleswarm' algorithm in Matlab R2024a was used for parameter optimization, with 'SwarmSize' set to 1000, 'MaxStallIterations' to 50 and 'HybridFcn' to @fmincon for constrained optimization, and otherwise default settings. The square root of the chi-squared error between model and data was used as the optimization criterion to be minimized. The average nuclear cross-sectional area of 2C cells (in pixels) was constrained to be in the range of areas in the dataset, and the scaling parameter was constrained to the range [1, 2.5]. The use of a scaling factor assumes that nuclear volume scales with ploidy (i.e. 4C nuclei are twice the size of 2C nuclei), and the range considers that cross-sectional areas may scale differently with ploidy depending on the (unknown) nuclear shape. If nuclei are approximately spherical for all ploidies, cross-sectional areas are expected to increase with a factor $2^{2/3} \cong 1.59$ when ploidy doubles. If nuclei on the other hand tend to expand more unidirectionally upon endoreplication (e.g. forming ellipsoids or cylinders along the length axis of expanding cells), the scaling factor may be closer to 2. 500 particle swarm optimization repeats were performed, resulting in an optimal solution with an average 2C nuclear cross-sectional area of 56.1525 pixels and a scaling factor of 1.6217, close to the scaling factor expected for spherical nuclei. Model code and data are available at https://github.com/maerelab/ploidyfit/ .

Flow cytometry

The endoploidy levels of WT and mutants (*sim*, *ccs52a1*, and *sim ccs52a1*) H cells at the 7-day time point were measured via flow cytometry using H file-specific marker lines holding a H2A-GFP tag. Collected root samples were chopped with a razor blade in 1 mL Galbraith's buffer (45 mM $MgCl_2$, 20 mM MOPS, 30 mM sodium citrate, 0.1 % Triton X-100, pH = 7.0 with NaOH) and filtered through a 30-µm CellTrics filter. The filtered supernatant was stained with 1 µL (1 mg/mL) DAPI. DAPI-stained nuclei were excited at 405 nm and equipped with an additional 488-nm laser to excite and detect GFP-specific fluorescence. The flow cytometric results were analyzed by FlowJo software.

Root hair measurements

Images of root hairs of 7-day-old seedlings were acquired by a VHX-7000 digital microscopy (Keyence). Parameters were quantified using ImageJ (Fiji version), with the distance between the root tip and the position of the first visible H cell being measured using the Straight-Line tool to draw a line from the root tip to the base of the H cell. Hair length measurements were performed on the first 20 visible hairs along the root axis, recorded in micrometers (µm) after scale calibration. The Segmented-Line tool traced each root hair from base to tip. To assess tip growth dynamics, 7-day-old seedlings were mounted on the VHX-7000 microscope stage within a stable imaging chamber to minimize disturbance. Time-lapse sequences were recorded over 5 hours with 5-min intervals. The hair growth rate was

quantified by manually tracing the position of hair tips in each frame using the Segmented-Line tool in ImageJ. The initial position (t = 0) was set as the baseline, and subsequent tip displacements were measured relative to this point. Growth rates were calculated as the total length increase (μm) divided by time (min).

Cross-section GUS staining

To investigate the expression pattern of *SIM* and *CCS52A1* in the WT primary root, cross-section staining was performed on β-glucuronidase-driven reporter lines of these two genes. Seedlings were fixed in ice cold 80% acetone for 30 min. After the washing in phosphate buffer, the seedlings were immersed in the enzymatic reaction mixture [0,5 mg/mL of 5-bromo-4-chloro-3-indolyl-beta-D-galactopyranoside; 0,5 mM ferricyanide and 0,5 mM ferrocyanide in 100 mM phosphate buffer (pH 7.4)]. The reaction was incubated at 37°C in the dark for 3 h. After washing in phosphate buffer, the seedlings were fixed in a paraformaldehyde (4%)/gluteraldehyde (1%) solution, dehydrated through a graded ethanol series, infiltrated in a Technovit 7100 (Heraeus Kulzer) series and embedded in Technovit 7100. Sections of 4-μm thickness were prepared and stained with 0,05% ruthenium red.

Material preparation for comprehensive microarray polymer profiling (CoMPP)

Roots were harvested at the 7-day time point and immediately frozen in liquid nitrogen. The freeze-dried materials were homogenized in 2mL Eppendorf tubes using TissueLyser LT (Qiagen). The finely homogenized lysate was mixed with 70 % ethanol, vortexed thoroughly, incubated for 30 min, and centrifuged at maximum speed for 10 min. This process was repeated three times. The pellet was collected and resuspended in acetone, followed by overnight air-drying to yield a whitish powder (=alcohol insoluble residue, AIR). The extraction weight was measured, with each sample weighing at least 5 mg.

Extraction of cell wall glycans

To extract the pectin-rich fractions from the alcohol insoluble residues (AIRs), 500 μL of 50 μM CDTA (pH 7.5) was added to each sample. After vortexing briefly, samples were centrifuged for 1 min at the max speed. The supernatant was carefully transferred into a new Eppendorf tube and stored at 4°C. The hemicellulose-rich fraction was sequentially extracted from the AIRs by incubating the washed pellet with 500 μL of 4 M NaOH containing 0.1% (w/v) $NaBH_4$. The supernatant was diluted a 1:2, 1:20, and 1:50 (v/v) in deionized water for the next step.

Printing microarrays, probing, and quantification

Arrays were printed on nitrocellulose sheets using an ArrayJet Sprint microarray printer (ArrayJet, Roslin, UK; Rydahl et al., 2018). Arrays were blocked [5 % skimmed milk powder in Tris-buffered saline

with Tween 20 (TBST): 20 mM Tris-HCl, 140 mM NaCl, pH 7.5, 0.1 % Tween 20] for 1 h, followed by incubation with primary cell wall antibodies or carbohydrate binding modules 1:100 in blocking solution for 1.5 h, followed by washing with TBST (3 x 5 min). Next, the secondary antibodies (anti-rat alkaline phosphatase conjugates; 1:1000 in blocking solution) were added and incubated for 1.5 h, followed by washing. Color was developed with BCIP/NBT substrate, and gray intensity of each colored precipitates was quantified.

Immuno-fluorescence labeling

Methylesterified homogalacturonan (HG) with a low degree of esterification (DE) was detected using an LM19 monoclonal antibody (PlantProbes). For *in situ* immuno-fluorescence, 7-day-old seedlings grown in channel slides (ibidi, 80601) were fixed in phosphate-buffered saline (PBS) containing 2.5 % (v/v) glutaraldehyde. Channels were then thoroughly rinsed with 3 mL of PBS applied with a syringe under constant pressure for 30 s to remove glutaraldehyde. Seedlings were then blocked in PBS with 2 % (w/v) BSA for 30 min, rinsed with a syringe, and then incubated with primary antibody LM19 (diluted 1:20 in PBS) at 22.5 °C for 2 h. After rinsing with PBS solution, a secondary antibody Alexa Fluor 488 goat anti-Rat IgM (heavy chain) cross-adsorbed (Invitrogen, A-21212) (diluted 1:50 in PBS) was added and incubated for 60 min at room temperature. Samples were rinsed with a syringe and subjected to confocal microscopy (Leica SP5; laser line 488 nm, EM 520–530 nm). This protocol was adapted from the procedure from Herburger et al. (2022).

RT-qPCR analysis

Total RNA was extracted from 5- to 10-mm distal root tips of 7-day-old Col-0 and *sim*, *ccs52a1*, and *sim ccs52a1* mutant seedlings using the RNA Miniprep kit (ReliaPrep). cDNA was synthesized using the qScript cDNA Supermix kit (Quantabio). Relative expression levels were determined with the LightCycler 480 Real-Time SYBR green PCR System (Roche). *PME/PMEI* genes were selected based on cell-type-specific gene expression generated by fluorescence-activated cell sorting (FACS) (Li et al., 2016; Shahan et al., 2022) and scRNA-seq data (https://rootcellatlas.org/). The *EMB2386* (At1G02780) and *PAC1* (At3G22110) reference genes were used for normalization. Primer sequences are listed in Supplemental Table S4.

PME extraction and activity assay

PME activities in crude extracts prepared from root tips of 7-day-old Col-0 and *sim*, *ccs52a1* and *sim ccs52a1* mutants were quantified using a colorimetric assay (Vivar-Vera et al., 2007; Xu et al., 2022). Root tips were frozen, ground to a fine powder, homogenized 1:10 (w/v) in 0.05% $Na_2S_2O_5$, centrifuged (10,000 g) for 10 min, and the pellet was resuspended 1:8 (w/v) in 0.05% $Na_2S_2O_5$ and centrifuged

again. The pellet was resuspended 1:8 (w/v) in 0.05% $Na_2S_2O_5$ containing 0.1% polyvinylpolypyrrolidone, centrifuged, resuspended in NaCl, and the pH adjusted to 7.5 using NaOH, followed by centrifugation and collection of the supernatant for the PME activity assay. The PME activity assay reaction mixture (100 µL) contained 15 µL crude PME extract, 0.1% (w/v) highly esterified pectin (Megazyme K-PECID; 1 mg/mL), and 0.01 U of *Pichia pastoris* alcohol oxidase (Sigma A2404) in 50 mM PBS (pH 7.5). After incubation (1 h at room temperature), the OD (420 nm) was measured, developing solution was added (2 mM ammonium acetate, 0.02 M pentane-2,4-dione, 0.05 M acetic acid), the mixture was incubated at 68°C for 15 min, and the OD measured again. Released nmol of methanol /min/µL was calculated using a methanol standard curve.

References

Decaestecker, W., Andrade Buono, R., Pfeiffer, M.L., Vangheluwe, N., Jourquin, J., Karimi, M., Van Isterdael, G., Beeckman, T., Nowack, M.K., and Jacobs, T.B. (2019). CRISPR-TSKO: a technique for efficient mutagenesis in specific cell types, tissues, or organs in Arabidopsis. Plant Cell **31,** 2868-2887.

Herburger, K., Schoenaers, S., Vissenberg, K., and Mravec, J. (2022). Shank-localized cell wall growth contributes to *Arabidopsis* root hair elongation. Nat. Plants **8,** 1222-1232.

Kurihara, D., Mizuta, Y., Sato, Y., and Higashiyama, T. (2015). ClearSee: a rapid optical clearing reagent for whole-plant fluorescence imaging. Development **142,** 4168-4179.

Li, S., Yamada, M., Han, X., Ohler, U., and Benfey, P.N. (2016). High-Resolution Expression Map of the Arabidopsis Root Reveals Alternative Splicing and lincRNA Regulation. Dev Cell **39,** 508-522.

Montenegro-Johnson, T.D., Stamm, P., Strauss, S., Topham, A.T., Tsagris, M., Wood, A.T.A., Smith, R.S., and Bassel, G.W. (2015). Digital single-cell analysis of plant organ development using 3DCellAtlas. Plant Cell **27,** 1018-1033.

Musielak, T.J., Bürgel, P., Kolb, M., and Bayer, M. (2016). Use of SCRI renaissance 2200 (SR2200) as a versatile dye for imaging of developing embryos, whole ovules, pollen tubes and roots. Bio-protocol **6,** e1935.

Shahan, R., Hsu, C.-W., Nolan, T.M., Cole, B.J., Taylor, I.W., Greenstreet, L., Zhang, S., Afanassiev, A., Vlot, A.H.C., Schiebinger, G., Benfey, P.N., and Ohler, U. (2022). A single-cell *Arabidopsis* root atlas reveals developmental trajectories in wild-type and cell identity mutants. Dev. Cell **57,** 543-560.

Strauss, S., Runions, A., Lane, B., Eschweiler, D., Bajpai, N., Trozzi, N., Routier-Kierzkowska, A.-L., Yoshida, S., Rodrigues da Silveira, S., Vijayan, A., Tofanelli, R., Majda, M., Echevin, E., Le Gloanec, C., Bertrand-Rakusova, H., Adibi, M., Schneitz, K., Bassel, G.W., Kierzkowski, D., Stegmaier, J., Tsiantis, M., and Smith, R.S. (2022). Using positional information to provide context for biological image analysis with MorphoGraphX 2.0. eLife **11,** e72601.

Tofanelli, R., Vijayan, A., Scholz, S., and Schneitz, K. (2019). Protocol for rapid clearing and staining of fixed Arabidopsis ovules for improved imaging by confocal laser scanning microscopy. Plant Methods **15,** 120.

Vivar-Vera, M.A., Salazar-Montoya, J.A., Calva-Calva, G., and Ramos-Ramírez, E.G. (2007). Extraction, thermal stability and kinetic behavior of pectinmethylesterase from hawthorn (*Crataegus pubescens*) fruit. Food Sci. Technol. **40,** 278-284.

Willems, A., Heyman, J., Eekhout, T., Achon, I., Pedroza-Garcia, J.A., Zhu, T., Li, L., Vercauteren, I., Van den Daele, H., van de Cotte, B., De Smet, I., and De Veylder, L. (2020). The Cyclin CYCA3;4 Is a Postprophase Target of the APC/C(CCS52A2) E3-Ligase Controlling Formative Cell Divisions in

Arabidopsis. Plant Cell **32,** 2979-2996.

Wolny, A., Cerrone, L., Vijayan, A., Tofanelli, R., Barro, A.V., Louveaux, M., Wenzl, C., Strauss, S., Wilson-Sánchez, D., Lymbouridou, R., Steigleder, S.S., Pape, C., Bailoni, A., Duran-Nebreda, S., Basse, G.W., Lohmann, I.U., Miltos, T., Hamprecht, F.A., Schneitz, K., Maizel, A., and Kreshuk, A. (2020). Accurate and versatile 3D segmentation of plant tissues at cellular resolution. eLife **9,** e57613.

Xu, F., Gonneau, M., Faucher, E., Habrylo, O., Lefebvre, V., Domon, J.-M., Martin, M., Sénéchal, F., Peaucelle, A., Pelloux, J., and Höfte, H. (2022). Biochemical characterization of Pectin Methylesterase Inhibitor 3 from *Arabidopsis thaliana*. The Cell Surface **8,** 100080.
